# Effect of *Mycobacterium leprae* on neurotrophins expression in human Schwann cells and mouse sciatic nerves

**DOI:** 10.1590/0074-02760200075

**Published:** 2020-07-17

**Authors:** Maria Renata Sales Nogueira, Nádia Ghinelli Amôr, Letícia Baccaro Michellin, Milton Cury, Patrícia Sammarco Rosa, Ana Carla Pereira Latini, Luciana Silva Rodrigues, Robertha Mariana Rodrigues Lemes, Flavio Alves Lara, Maria Cristina Vidal Pessolani

**Affiliations:** 1 Secretaria de Estado da Saúde de São Paulo, Instituto Lauro de Souza Lima, Bauru, SP, Brasil; 2 Universidade de São Paulo, Faculdade de Odontologia de Bauru, Departamento de Ciências Biológicas, Bauru, SP, Brasil; 3 Universidade do Estado do Rio de Janeiro, Laboratório de Imunopatologia, Rio de Janeiro, RJ, Brasil; 4 Fundação Oswaldo Cruz-Fiocruz, Instituto Oswaldo Cruz, Laboratório de Microbiologia Celular, Rio de Janeiro, RJ, Brasil

**Keywords:** leprosy, Schwann cells, neurotrophins

## Abstract

**BACKGROUND:**

Although *Mycobacterium leprae* (ML) is well characterised as the causative agent of leprosy, the pathophysiological mechanisms underlying peripheral nerve damage still need further understanding. *In vitro* and *in vivo* studies have yielded insights into molecular mechanisms of ML interaction with Schwann cells (SC), indicating the regulation of genes and proteins crucial to neural plasticity.

**OBJECTIVES:**

We aimed to investigate the effect of ML on neurotrophins expression in human SC (hSC) and mice sciatic nerves to better understand their role in leprosy neuropathy, and aiming to contribute to future therapeutic approaches.

**METHODS:**

We evaluated mRNA and protein expression of BDNF, NGF, NT-3, NT-4 in hSC from amputation nerve fragments, as well as in athymic nude mice, infected by ML for eight months.

**FINDINGS and MAIN CONCLUSIONS:**

Our *in vitro* results showed a trend to decline in NGF and BDNF mRNA in ML-treated hSC, compared to controls. The immunodetection of BDNF and NT-4 was significantly downregulated in ML-treated hSC. Conversely, ML-infected mice demonstrated upregulation of NT-3, compared to non-infected animals. Our findings indicate that ML may be involved in neurotrophins regulation, suggesting that a pathogen-related imbalance of these growth factors may have a role in the neural impairment of leprosy.

Leprosy-associated neuropathy is triggered by *Mycobacterium leprae* (ML), an obligate intracellular pathogen that invades the host via the upper airways, disseminates hematogenously, and leads to an asymmetrical pattern of infection in peripheral nerves.^1^ The tropism of ML by peripheral nerves has been initially attributed to its binding to Schwann cells (SC) surface throughout a complex formed by alpha-dystroglycan receptors and the extracellular matrix protein, laminin-α2, associated with the basal lamina of SC.^2^

Although ML has been recognised as the causative agent of leprosy for more than a century, pathophysiological mechanisms underlying nerve damage still need further understanding.^3^ SC supports and myelinates axons and, once infected, ML induces dedifferentiation, proliferation, and reprogramming these glial cells, regulating genes and proteins crucial to neural plasticity that, in turn, lead to demyelination, axonal atrophy and physical disabilities.^3^

Neural development, maintenance and repair require a range of molecular key players including the neurotrophin family of growth factors. Neurotrophins are produced by nerves, cells with astroglial differentiation, fibroblasts, and leukocytes. In mammals, four major growth factors have been identified in the neurotrophin family: nerve growth factor (NGF), brain-derived neurotrophic factor (BDNF), neurotrophin-3 (NT-3) and neurotrophin-4 (NT-4).^4^

Nerve growth factor (NGF) was the first well-characterised neurotrophin, highlighted by its regulatory functions in differentiated neurons and other neural tissues.^5^ Interestingly, tissue sources of NGF (and other neurotrophins) in the periphery are typically nonneuronal cells.^6^ The second neurotrophic factor isolated was brain-derived neurotrophic factor (BDNF), which revealed structural similarity to NGF, leading to the concept of the neurotrophin family.^7^ Later studies led to the identification of two additional members of this family, namely, neurotrophin-3 (NT-3) and neurotrophin-4 (NT-4).^8^

Seminal studies on the role of neurotrophins in leprosy have sought to understand the implication of ML infection on the maintenance of host cells neurotrophic responses.^9^ ^,^ ^10^ Singh et al. evaluated possible alterations in the expression of NGF and the receptor p75 (p75NTR) after infection of murine SC; these parameters were compared in two mice strains with different responses to the bacillus: Swiss White, as a susceptible strain and C57Bl/6, as a resistant strain.^9^ The results demonstrated that the production of NGF did not change significantly after infection, while the expression of p75NTR decreased in both strains, suggesting that the neurotrophic loss could occur due to the deficiency of p75NTR instead of the deficiency of the ligand NGF.^9^

In contrast, the decrease in endogenous levels of NGF and the TrKA receptor was demonstrated directly in skin lesions of leprosy patients and correlated with nociceptive changes.^10^ Chan et al. verified that exogenous NGF enhances SC myelination, and the inhibition of NGF in peripheral nerves impairs neuroregeneration after injury, highlighting the role of NGF in leprosy-associated neuropathy.^11^ Studies evaluating BDNF, and NT3/4 levels during leprosy are remarkably scarce and not updated, but their role in other neuropathy have been explored and allow the speculation on their impact on leprosy. Nerve transection leads to a marked increase in BDNF expression in murine SC, suggesting its role in nerve repair.^12^ One study demonstrated that BDNF levels tend to be lower in leprosy patients than in healthy individuals, which might reflect how ML-infection triggers the loss of neurotrophic stimulus by BDNF downregulation.^13^

The therapeutic implications in neurotrophins regulation on the myelination program of the peripheral nervous system may be extended to demyelinating peripheral neuropathies, including leprosy, and nerve injury.^14^ Given the significant role of neurotrophins on neural homeostasis and recovery after injury, we aimed to investigate the effect of ML on neurotrophins expression in human SC and mice sciatic nerves to better understand their role in leprosy neuropathy, and aiming to contribute to future therapeutic approaches.

## SUBJECTS AND METHODS

*Human* - The participants of the study were selected at the outpatient clinic of Lauro de Souza Lima Institute, São Paulo State Health Secretariat, Bauru, southeastern Brazil. Individuals included were between 18 and 60 years of age, of both sexes, with the indication for surgical amputation due to trauma in the upper or lower limbs. Seven human nerve fragments were collected. Patients with previous peripheral neuropathies were not included in this study. Procedures were under the ethical standards of the Human Ethics Committee of the Lauro de Souza Lima Institute (protocol # 185/09), and the Helsinki Declaration (1964). All participants were informed about the aims of the study, and the procedures involved. Participants were included after the Informed Consent Form signing.

*Animals* - *In vivo* experiments were conducted in 15 athymic *nude* mice (NU-*Foxn1* ^*nu*^ ), with 60 days old, from the central animal facilities in Lauro de Souza Lima Institute. The death of the animals was induced by an overdose of ketamine 200 mg/kg (Vetnil) and Rompun® 30 mg/kg (Bayer) intraperitoneally. The handling of the experimental animals was under the Ethics Committee on Animal Teaching and Research, from Bauru School of Dentistry, University of São Paulo, Bauru, Brazil (process nº 021/2010).

*Schwann cells culture* - Human nerves were collected in a surgical centre and stored in appropriated transport medium. Each nerve had, on average, 2.5 cm x 0.2 cm x 0.3 cm in its largest dimensions. In the laminar flow hood, the specimens were fragmented with a sterile scalpel, transferred to a conical tube with phosphate-buffered saline (PBS), and centrifuged at 1200 rpm for 5 min, at 4ºC. Fragments were resuspended in Dulbecco’s Modified Eagle’s Medium (DMEM-High glucose) (Gibco®) with 10% foetal bovine serum (FBS) (Sigma-Aldrich), and supplemented with 10 ng/mL β-heregulin (HRG) (Sigma-Aldrich), 2 μM forskolin (Sigma-Aldrich) and 1% penicillin/streptomycin (Gibco®). Human nerve fragments were kept in explant for seven days, in 5% CO_2_ incubator with 95% humidity, at 37ºC. The specimens were then centrifuged at 1200 rpm for 5 min at 4ºC, resuspended in DMEM-Hg containing 1% penicillin/streptomycin, 0.5 mg/mL collagenase type I and 2.5 mg/mL dispase II (Gibco®), and maintained in a CO_2_ incubator for 24 h. Human SC suspension (hSC) was transferred to DMEM-Hg/10% FBS and centrifuged at 1500 rpm for 10 min at 4ºC. Pellet was resuspended for cell counting and trypan blue viability exclusion. hSC were seeded and expanded up to 80% of confluence in polystyrene 6-well plates, coated with 20 μg/mL laminin (Sigma-Aldrich). After reaching confluence, cell passage was performed, and cell densities were adjusted to 2.5 x 10^4^ cells/cm^2^ in 24-well plates. The experimental groups were then incubated with sonicated ML (sML, Colorado State University, Fort Collins, Colorado, USA) at 10 μg/mL and maintained for 24 and 48 h. The control group was not exposed to sML. After the experimental period, hSC was fixed in 4% paraformaldehyde (PFA) (Sigma-Aldrich), washed in PBS, and stored at -20ºC until evaluation.

*M. leprae* - Mouse infection was conducted after harvesting viable ML (vML) (Thai-53 strain) from serial passages in the footpads of athymic *nude* mice (NU-*Foxn1* ^*nu*^ ), according to the previously described technique.^15^ Briefly, the animals were inoculated into the plantar surface of both hind footpads with 30 μL of the bacillary suspension containing 1 x 10^7^ bacilli/mL. After four months, the animals were killed and the bacillary suspension was prepared.

*Mouse inoculation* - Inoculation was performed by intradermal injection with 100 μL of vML suspension at 1 x 10^6^ bacilli/mL in the popliteal fossa, near to the sciatic nerve trifurcation, bilaterally. The popliteal fossa was chosen due to the notion that this is an adequate access point for the anesthetic sciatic nerve blockade. An additional 30 μL of the same suspension was inoculated into hind footpads of each mouse. Mice were maintained for eight months (N = 5). Subsequently, mice were killed, and both sciatic nerves collected to the same evaluations conducted in hSC culture. Nerves were fixed in 4% PFA (Sigma-Aldrich) for 15 min and incubated overnight in 30% sucrose at 4ºC, being later prepared for longitudinal cryosections. Sciatic nerves of non-infected nude mice (N = 5) were adopted as controls.

*Antibodies* - Anti-BDNF (1:100, Abcam), anti-NGF (1:250, Millipore), anti-NT-3 (1:100, Millipore), anti-NT-4 (1:100, Millipore), anti-p75NTR (1:100, R&D Systems), anti-S100β (1:1000, Sigma-Aldrich), anti-MPZ (1:100, Abcam) and anti-NF-L (1:100, Dako).

*Immunofluorescence* - Assays were performed in hSC cultures and mouse sciatic nerves. The samples were incubated with primary antibodies overnight, then incubated with anti-mouse or anti-rabbit fluorochrome-conjugated secondary antibodies AlexaFluor 488 and AlexaFluor 594 (1:400, Molecular Probes), for 60 min. Nuclei were stained with 4’,6-diamidino-2-phenylindole (DAPI. 1:200, Molecular Probes), and coverslips were mounted with Slow Fade Anti-Fading Kit (Molecular Probes). The image capture was performed in a confocal laser scanning microscope TCS SP5 (Leica, Germany). Image analysis was performed in ImageJ software (National Institutes of Health, USA), in which fluorescence was converted into values of integrated density. In cell culture assays, data of integrated density were normalised by the number of DAPI^+^ nuclei in each field.

*Enzyme-linked immunosorbent assay (ELISA)* - Cell culture supernatants were collected from hSC, treated or not by sML. BDNF analysis was performed by ChemiKine BDNF Sandwich ELISA kit (Millipore, MA, USA), NGF and NT-3 were analysed by Human ELISA Kits (Abcam, MA, USA), according to the manufacturer’s recommendations. Briefly, BDNF, NGF and NT-3 assays proceeded as follows: 100 μL of each standard and sample were added into appropriate wells and incubated overnight, at 4ºC. The solution was discarded and wells were rinsed four times with wash solution. Next, it was added 100 μL of biotinylated beta NGF and NT-3 detection antibodies to each well and incubated for 1 h at room temperature (RT). After solution discard, 100 μL of HRP-Streptavidin was added and incubated for 45 min, at RT. Following new washes and discard, 100 μL of TMB was added and incubated for 30 min, at RT in the dark. Finally, 50 μL of stop solution was added to each well. Optical density (OD) was analysed at 450 nm immediately. All samples were analysed in duplicate. NT-4 detection was not performed because the concentration of this neurotrophin is pretty low in serum/plasma, and may not be detected in this assay.

*RNA Extraction* - Specimens from hSC culture were suspended in Trizol (Invitrogen, USA). Subsequently, 0.2 mL of chloroform was added to each mL of suspension. The samples were centrifuged and the aqueous phase transferred to a new tube, to which the same volume of isopropanol was added. The tubes were centrifuged again, the precipitate was washed in 100% ethanol, and dried at room temperature. The RNA samples were suspended in deionised water, free of RNAse, and stored at -80ºC. An aliquot was used to obtain RNA/μL concentration in each specimen. The complementary DNA (cDNA) was synthesised through a reverse transcription reaction using 1 μg of RNA.

*Reverse transcription-polymerase chain reaction (RT-PCR)* - Quantification of mRNA transcribed from BDNF, NGF and NT-3 genes was carried out with TaqMan-type assays (Applied Biosystems), according to the protocol for the Step One Plus equipment (Applied Biosystems). The analysis of neurotrophins expression by RT-PCR was performed by relative quantification, using GAPDH as the endogenous control.

*Statistical analysis* - Results were analysed in GraphPad Prism 7.04 (GraphPad Software Inc., USA). Shapiro-Wilk normality test was applied to determine the choice of parametric or non-parametric evaluations. Unpaired t-test with Welch’s correction was applied for two-group comparisons. When more than two groups were compared, Kruskal-Wallis test with Dunn’s multiple comparisons test was performed. P values < 0.05 were considered as the cutoff for significance.

## RESULTS

**Fig. 1:**
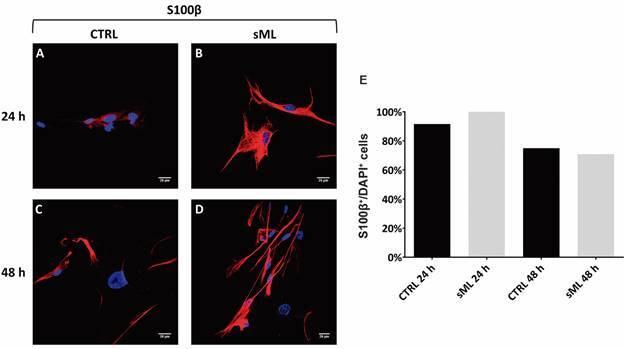
immunophenotypic characterisation of human Schwann cells. Cells were obtained from healthy donors and cultivated for 24 and 48 h alone (CTRL) or in the presence of sonicated *Mycobacterium leprae* (sML). (A-D) Confocal microscopy showing immunodetection of S100β protein (red, AlexaFluor 594, Molecular Probes) and nuclear staining by DAPI (blue). Scale bar = 20 µm. (E) Graph shows the mean of the percentage of S100β^+^/DAPI^+^ cells.

**Fig. 2:**
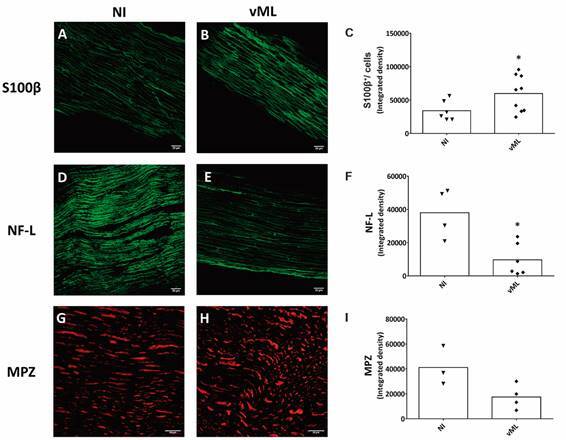
immunophenotypic characterisation of mouse sciatic nerves. Sciatic nerves were resected from healthy nude mice non-infected (NI) or mice inoculated with viable *Mycobacterium leprae* (vML) during eight months. Nerves were cryopreserved and incubated with the following primary antibodies and corresponding secondary antibodies: S100β (green, AlexaFluor 488, Molecular Probes); NF-L (green, AlexaFluor 488, Molecular Probes), and MPZ (red, AlexaFluor 594, Molecular Probes). Confocal images illustrate fluorescent detection of S100β (A-B), NF-L (D-E) and MPZ (G-H). Scale bar = 20 µm. (C, F, I) Graphs showing scatter plots with mean values, obtained from unpaired t-test with Welch’s correction. *p < 0.05. Each dot in scatter plots represents nerve fragments, one per animal. Immunofluorescence was performed in 3-6, and 4-9 nerve fragments in NI and vML-infected mice, respectively.

*Characterisation of Schwann cells and sciatic nerves* - First, hSC were immunophenotypically characterised, and the presence of possible perineural fibroblasts, considered as contaminants, was quantified. The purity of the hSC culture was verified by the ratio between S100β^+^/DAPI^+^ cells (SC) *versus* S100β^-^/DAPI^+^ cells (non-glial cells) (Fig. 1A-D). Non-glial cells were approximately 12% (Fig. 1E), and there was no difference between control (CTRL) and sML-treated cells. Thereby, treatment with sML for 24 and 48 h does not seem to affect hSC immunophenotypically. Sciatic nerves were characterised by S100β, NF-L, and MPZ positive immunostaining (Fig. 2A-I). Non-infected (NI) and vML-infected nerves were compared. The immunodetection of S100β was higher in the vML group than NI (Fig. 2B-C), whereas NF-L was lower in vML (Fig. 2E-F), compared with the NI group. No significant differences were observed regarding MPZ immunodetection (Fig. 2G-I).

**Fig. 3:**
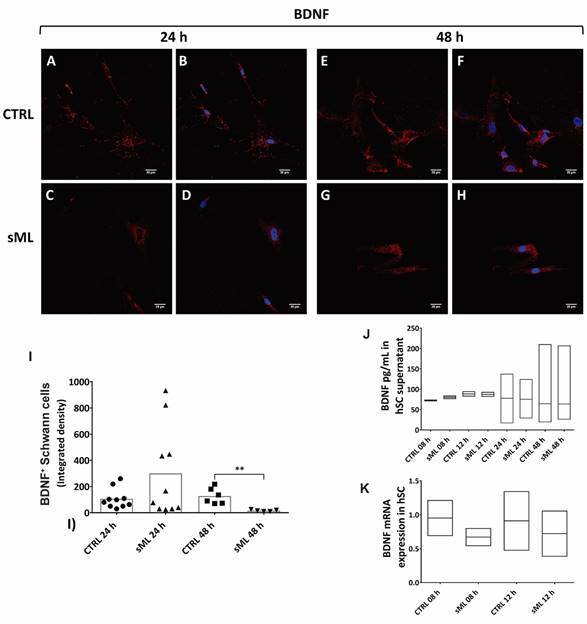
BDNF in human Schwann cells (hSC). Confocal microscopy of BDNF immunodetection (red, AlexaFluor 594, Molecular Probes) and nuclear labeling by DAPI (blue, Molecular Probes) in hSC culture, treated or not with sonicated *Mycobacterium leprae* (sML, 10 µg/mL) for (A-D) 24 and (E-H) 48 h. Scale bar = 20 µm. (I) Graph illustrates the fluorescence intensity of BDNF in hSC, shown as scatter plots with means, from unpaired t-test with Welch’s correction. ** p < 0.01. (J) The secretion of BDNF is shown in the graph of hSC culture supernatants treated by sML for 08, 12 and 24 h. BDNF concentration (pg/mL) was retrieved from four independent assays. Kruskal-Wallis test with Dunn’s multiple comparisons test (p > 0.05), line at the median. (K) BDNF mRNA expression, normalised by GAPDH, is graphically demonstrated in unstimulated (CTRL) and sML-treated hSC, after 08 and 12 h. Reverse transcription-polymerase chain reaction (RT-PCR) results from two independent assays. Kruskal-Wallis test with Dunn’s multiple comparisons test (p > 0.05), line at the median.

**Fig. 4:**
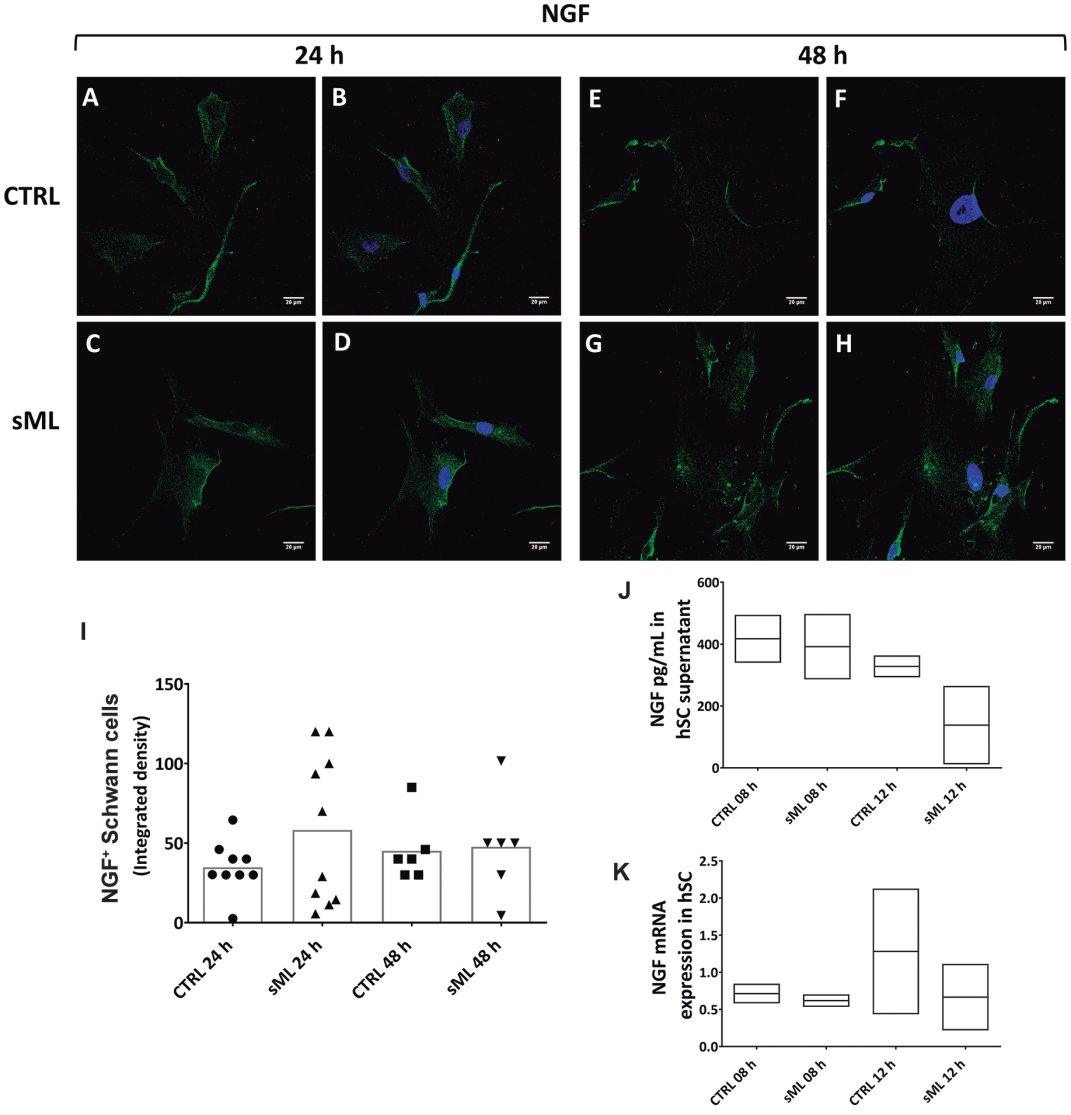
NGF in human Schwann cells (hSC). Confocal microscopy of NGF immunodetection (green, AlexaFluor 488, Molecular Probes) and nuclear labeling by DAPI (blue, Molecular Probes) in hSC culture treated or not with sonicated *Mycobacterium leprae* (sML, 10 µg/mL) for (A-D) 24 and (E-H) 48 h. Scale bar = 20 µm. (I) Graph illustrates the fluorescence intensity of NGF in hSC, shown as scatter plots with means, from unpaired t-test with Welch’s correction. (J) The secretion of NGF is shown in graphs of hSC culture supernatants treated by sML for 08 and 12 h. NGF concentration (pg/mL) was retrieved from two independent assays. Kruskal-Wallis test with Dunn’s multiple comparisons test (p > 0.05), line at the median. (K) NGF mRNA expression, normalised by GAPDH, is graphically demonstrated in unstimulated (CTRL) and sML-treated hSC, after 08 and 12 h. Reverse transcription-polymerase chain reaction (RT-PCR) results from two independent assays. Kruskal-Wallis test with Dunn’s multiple comparisons test (p > 0.05), line at the median.

**Fig. 5:**
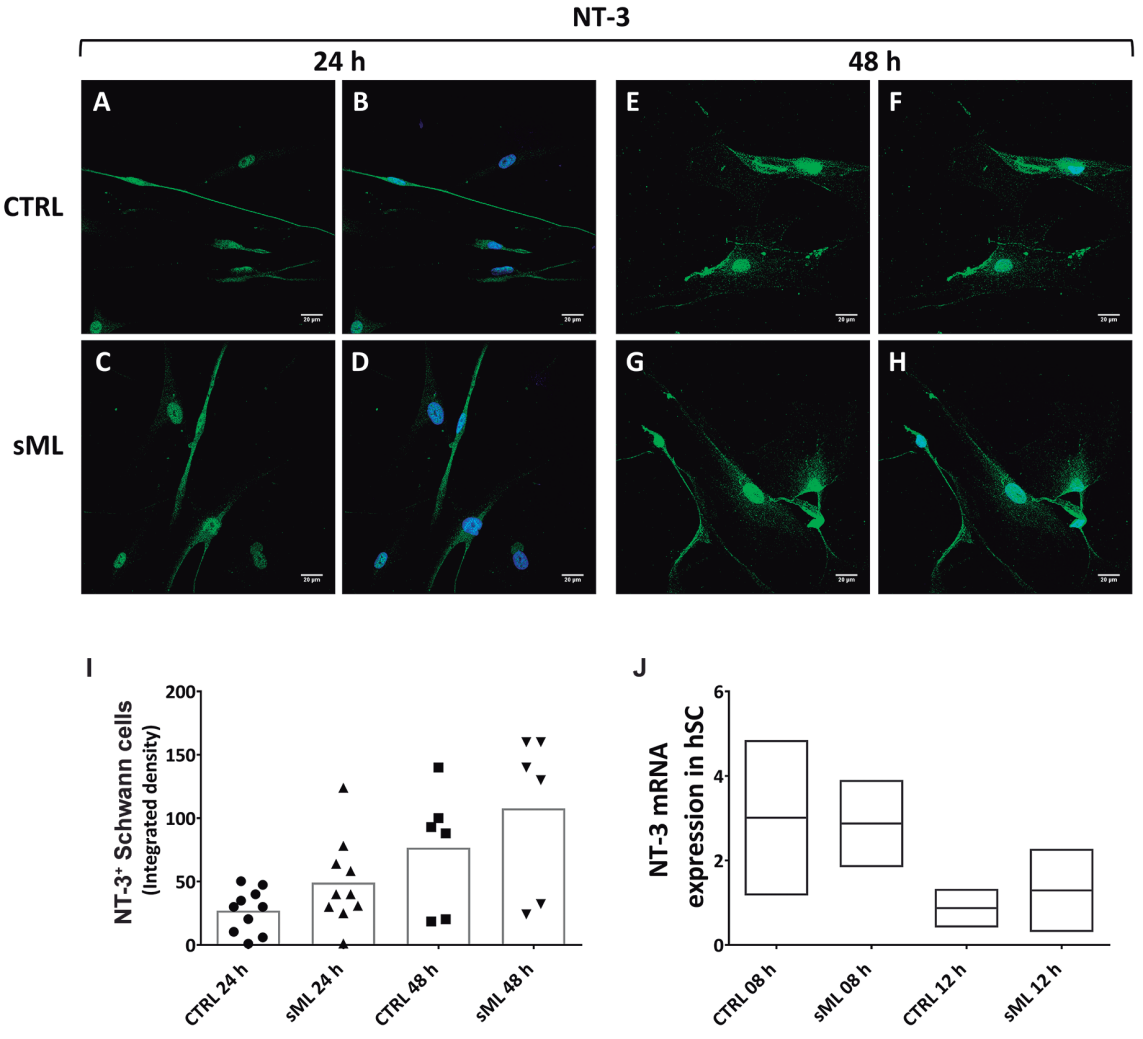
NT-3 in human Schwann cells (hSC). Confocal microscopy of NT-3 immunodetection (green, AlexaFluor 488, Molecular Probes) and nuclear labeling by DAPI (blue, Molecular Probes) in hSC culture treated or not with sonicated *Mycobacterium leprae* (sML, 10 µg/mL) for (A-D) 24 and (E-H) 48 h. Scale bar = 20 µm. (I) Graph illustrates the fluorescence intensity of NT-3 in hSC, shown as scatter plots with means, from unpaired t-test with Welch’s correction. (J) NT-3 mRNA expression, normalised by GAPDH, is graphically demonstrated in unstimulated (CTRL) and sML-treated hSC, after 08 and 12 h. Reverse transcription-polymerase chain reaction (RT-PCR) results from two independent assays. Kruskal-Wallis test with Dunn’s multiple comparisons test (p > 0.05), line at the median.

**Fig. 6:**
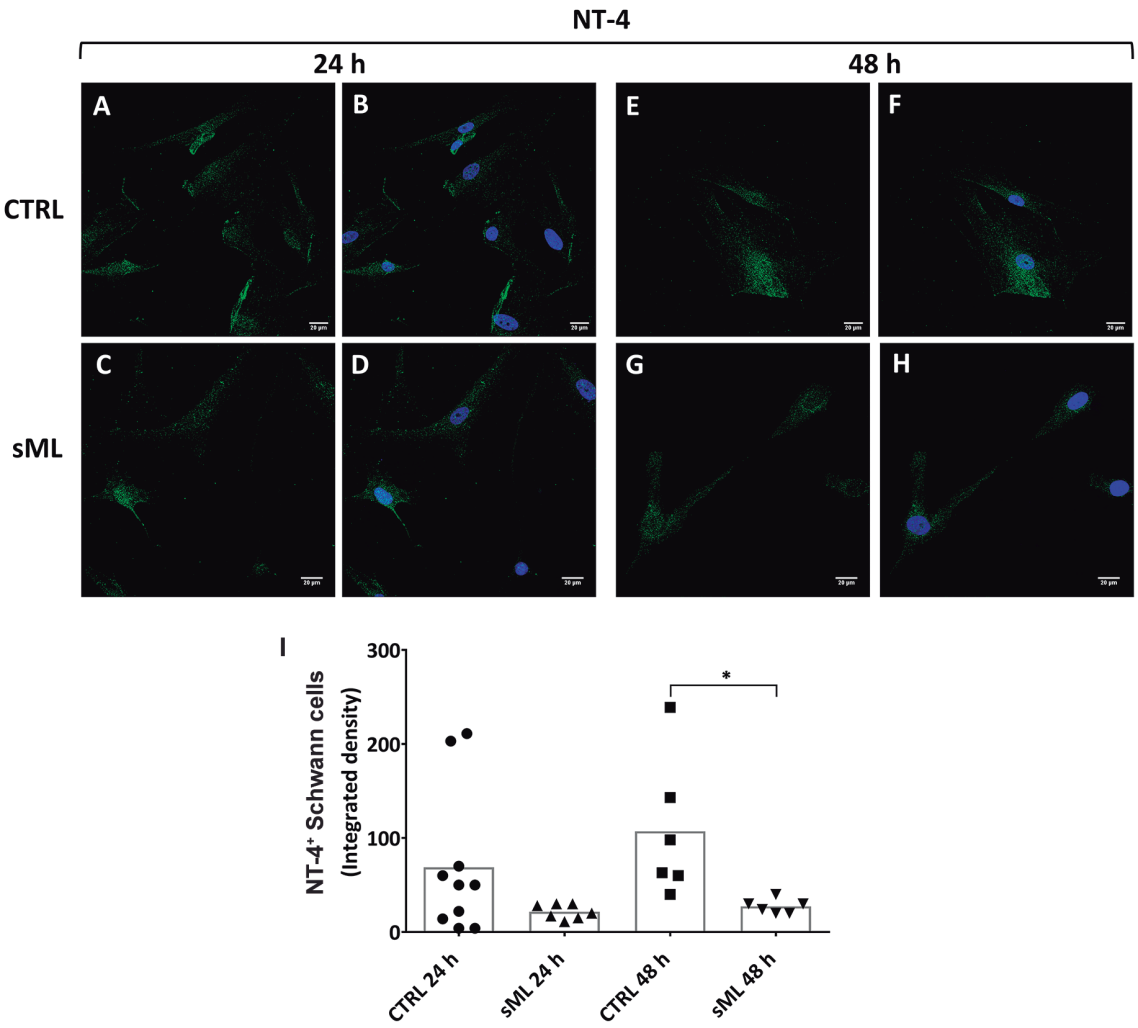
NT-4 in human Schwann cells (hSC). Confocal microscopy of NT-4 immunodetection (green, AlexaFluor 488, Molecular Probes) and nuclear labeling by DAPI (blue, Molecular Probes) in hSC culture treated or not with sonicated *Mycobacterium leprae* (sML, 10 µg/mL) for (A-D) 24 and (E-H) 48 h. Scale bar = 20 µm. (I) Graph illustrates the fluorescence intensity of NT-4 in hSC, shown as scatter plots with means, from unpaired t-test with Welch’s correction. * p < 0.05.

*sML treatment decreases BDNF and NT-4 in human Schwann cells in vitro* - Next, we pursue to evaluate the effects of sML treatment on neurotrophins expression and production in hSC culture by immunofluorescence, ELISA and RT-PCR. Although sML treatment did not affect the expression of BDNF in the first 24 h (Fig. 3A-D), after 48 h BDNF was significantly decreased in sML-treated hSC, compared to CTRL (Fig. 3E-H). sML had no expressive effects on the expression of NGF (Fig. 4), neither on NT-3 (Fig. 5) in both periods analysed, 24 and 48 h. Similar to BDNF, the expression of the NT-4 was not affected by sML in the early period (Fig. 6A-D); however, sML-treated hSC had a significant decrease in NT-4 expression after 48 h of culture, compared with CTRL (Fig. 6E-H). The secretion of BDNF, NGF and NT-3 was analysed in hSC culture supernatants, stimulated or not by sML, after 8, 12 and 24 h. ELISA results retrieved low concentrations of NT-3, below the standard curve (Fig. 5J). The interpolated values of BDNF (Fig. 3J) and NGF (Fig. 4J), indicated a trend to decay in sML groups compared to controls, although without significant differences. Neurotrophins mRNA expression was also investigated in unstimulated *versus* sML-treated hSC culture, after 08 and 12 h. The analysis was performed by the relative standard curve method, in which the endogenous control was GAPDH. BDNF (Fig. 3K) and NGF (Fig. 4K) presented a trend to decrease in sML-treated cells, compared to controls. On the other hand, NT-3 demonstrated a discrete increase at 12 h, in sML-treated cells *versus* controls (Fig. 5K).

**Fig. 7:**
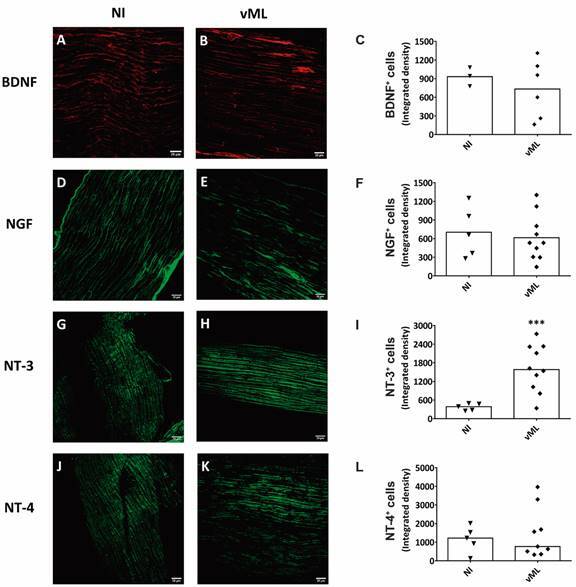
neurotrophins expression in mouse sciatic nerves. Confocal images and respective graphs illustrating the immunodetection of (A-C) BDNF (red, AlexaFluor 594, Molecular Probes), (D-F) NGF (green, AlexaFluor 488, Molecular Probes), (G-I) NT-3 (green, AlexaFluor 488, Molecular Probes), and (J-L) NT-4 (green, AlexaFluor 488, Molecular Probes) in uninfected (NI) and viable ML-infected mice (vML. 1 x 10^6^ bacilli/mL) over eight months. Scale bar = 20 µm. Graphs are shown as scatter plots with mean (C, F, I) and medians (L), from unpaired t-test with Welch’s correction and Mann Whitney test, respectively. *** p < 0.005. Each dot in scatter plots represents nerve fragments, one per animal. Immunofluorescence was performed in 3-5, and 6-10 nerve fragments in NI and vML-infected mice, respectively.

*NT-3 is increased in mouse sciatic nerves after vML infection* - Finally, we evaluated by immunofluorescence the expression of neurotrophins *in vivo*, in athymic nude mice (Fig. 7). Three neurotrophins, BNDF, NGF, and NT-4, displayed a discreet decrease in their expression on vML-infected mice, compared with NI (Fig. 7A-F, J-L). However, NT-3 expression was significantly higher in vML-infected mice than NI animals (Fig. 7G-I).

## DISCUSSION

Progressive demyelination in nerves infected by ML has been previously verified *in vivo* and *in vitro.* Glial pathology is a leprosy feature shared by other peripheral neuropathies, such as Guillain-Barré and Charcot-Marie-Tooth syndromes, multiple sclerosis, and chronic nerve compression injury.^16^ Likewise, the idea of demyelination as a pathological manifestation, rather than the etiology of nerve injury, has gained traction among some of the peripheral neuropathies, being currently discussed in leprosy.^17^ Multiple neurotrophin-related signaling pathways are involved in nerve damage and repair and the role of neurotrophins in leprosy have already been approached, mainly regarding two neurotrophins, NGF and BDNF.^18^ ^,^ ^19^ To our knowledge, this is the first study that describes the effects of ML on the expression of all four members of the neurotrophin family ― NGF, NT-3, NT-4, BDNF ― in hSC culture, as well as in sciatic nerves of athymic nude mice. Here we demonstrated a trend to decline in NGF and BDNF mRNA expression in ML-treated hSC, compared to controls. The immunodetection of BDNF and NT-4 was significantly downregulated in ML-treated hSC. Conversely, ML-infected mice demonstrated significant upregulation of NT-3, compared to non-infected animals. Our findings indicate that ML may be involved in neurotrophins regulation.

Before *in vitro* experiments, part of the hSC was submitted to immunophenotypic characterisation by S100β immunostaining, along with the identification of perineural fibroblasts. It was noted that the non-glial contaminating cells composed approximately 12% of the cultivated cells; though, a 5% maximum is preferred. To improve hSC culture purification, cell sorting techniques should be applied, such as magnetic sorting or fluorescence-activated cell sorting (FACS). Yet, we considered 12% an acceptable value for this kind of cell culture. Morphologically, hSC culture maintained typical tripolar or fusiform patterns, and the detection of S100β did not show significant differences when ML-treated hSC was compared to controls. Mice ML-infection resulted in increased immunostaining for S100β and a decrease of the axonal marker, NF-L. S100β is a well-established SC marker.^20^ Since remyelination depends upon the proliferation, migration, and differentiation of SC, S100β increase observed in our animal model, might reflect an attempt to revert the infection injury due to ML by increasing the presence of SC.^21^ On the other hand, the immunodetection of NF-L is axon-dependent thereby being downregulated during axonal atrophy.^22^

The relevance of BDNF in peripheral nerve injury and neuropathic pain was highlighted in studies on diabetic peripheral neuropathy.^23^ The decreased in serum levels of BDNF and NGF has been demonstrated in patients with diabetic peripheral neuropathy, suggesting that multiple deficits in these neurotrophic factors could precede clinically the detectable nerve dysfunction.^23^

The decreased of cytoplasmic expression and secretion of BDNF in hSC treated with sML could be associated with a declined neurotrophic and immunomodulatory character of hSC, hampering the tissue integrity. The neuroprotective role of SC-derived BDNF was demonstrated by Hou et al. study, in which BDNF-enriched SC enhanced *in vitro* its proliferative and secretory functions.^24^ The authors also have shown that, in an animal model of nerve damage, the transplantation of BDNF-enriched SC to the injury sites was able to reduce the inflammatory process and promoted neural repair.^24^ In our study, the decrease of BDNF in nude mice infected by vML for eight months, suggests that the availability of these growth factors become deficient in the long turn, failing to sustain the plasticity of peripheral nerves affected by leprosy.

Successful nerve regeneration requires axon regrowth and remyelination, and neurotrophins have a central role in supporting SC migration and myelination.^25^ Yin et al. have shown that NT-4 delivery increases the expression of myelin-associated glycoprotein (MAG) in SC, contributing to the functional recovery in the murine model of sciatic nerve transection.^26^ Additionally, the downregulation of NT-4 in experimental diabetes seems to be involved in the development and maintenance of diabetic neuropathy.^27^ Rodríguez-Peña et al. demonstrated that the expression of NT-4 was decreased to 29%, after 12 weeks of streptozotocin-induced diabetes in rat sciatic nerves.^27^ In accordance, the reduction of NT-4 in sML-treated hSC verified in our study suggests a possible role of this neurotrophin in SC function and leprosy neuropathy. To our knowledge, the present findings regarding NT-4 in sML-treated hSC are demonstrated for the first time.

Regardless of our *in vitro* evidence on neurotrophins regulation in sML-treated hSC, we faced sample size limitations due to the restricted availability of human nerve fragments. To corroborate our present findings, we judge opportune to replicate our experiments with a larger number of specimens. To overcome the sample size issue, an alternative source of hSC could come from glial cells differentiated from human hair follicles and dermis.^28^ ^,^ ^29^

Our *in vivo* data revealed that the expression of NT-3 was significantly higher in vML-infected mice than non-infected animals, an opposite result to the other neurotrophins evaluated here. Previous pieces of evidence from animal models of peripheral neuropathy depicts the effects of NT-3 on myelination.^14^ ^,^ ^30^ Liu et al. investigated the role of the NT3-TrkC pathway in myelination of Trembler-J mouse, a model of Charcot-Marie Tooth 1A (CMT1A), and observed that the injection of NT-3 decreased the myelin protein zero (P0) level in sciatic nerves.^30^ Chan et al. demonstrated *in vitro* and *in vivo*, that the addition of exogenous NT-3 inhibited myelin formation and the removal of the endogenous NT-3 enhanced myelination.^14^

The *in vivo* infection in our study was performed in sciatic nerves through inoculation of vML (1 x 10^6^ bacilli/mL) inside the popliteal fossa, near to the sciatic nerve trifurcation, and into hind footpads of each mouse. Our purpose was to allow bacillary traffic to endoneurial space, without forcing it inside the neural environment. However, after eight months post-inoculation solely perineural infection has been achieved (data not shown). Still, our data indicate a possible upregulation of NT-3 in vML-infected mice, suggesting that NT-3 could contribute to demyelination during ML infection. Future *in vivo* studies, designed with inoculation of ML directly into the endoneurial environment of murine sciatic nerves, could bring additional understanding to the early neural response to this pathogen in the regulation of neurotrophic factors.

Taken together, our findings indicate that ML may be involved in neurotrophins regulation, *in vitro* and *in vivo*, suggesting that a pathogen-related imbalance of these growth factors may have a role in the neural impairment of leprosy, reinforcing the need of further investigations.

## References

[1] Scollard DM, Truman RW, Ebenezer GJ (2015). Mechanisms of nerve injury in leprosy. Clin Dermatol.

[2] Rambukkana A, Yamada H, Zanazzi G, Mathus T, Salzer JL, Yurchenco PD (1998). Role of alpha-dystroglycan as a Schwann cell receptor for Mycobacterium leprae. Science.

[3] Hess S, Rambukkana A (2019). Cell biology of intracellular adaptation of Mycobacterium leprae in the peripheral nervous system. Microbiol Spectr.

[4] Allen SJ, Dawbarn D (2006). Clinical relevance of the neurotrophins and their receptors. Clin Sci (Lond).

[5] Levi-Montalcini R (1987). The nerve growth factor 35 years later. Science.

[6] Skaper SD (2018). Neurotrophic factors An overview. Methods Mol Biol.

[7] Leibrock J, Lottspeich F, Hohn A, Hofer M, Hengerer B, Masiakowski P (1989). Molecular cloning and expression of brain-derived neurotrophic factor. Nature.

[8] Ibanez CF (1995). Neurotrophic factors from structure-function studies to designing effective therapeutics. Trends Biotechnol.

[9] Singh N, Birdi TJ, Antia NH (1997). Nerve growth factor production and expression of p75 by Schwann cells and neurofibroblasts in response to M leprae infection and macrophage secretory products. Neuropathol Appl Neurobiol.

[10] Facer P, Mann D, Mathur R, Pandya S, Ladiwala U, Singhal B (2000). Do nerve growth factor-related mechanisms contribute to loss of cutaneous nociception in leprosy. Pain.

[11] Chan JR, Watkins TA, Cosgaya JM, Zhang CZ, Chen L, Reichardt LF (2004). NGF controls axonal receptivity to myelination by Schwann cells or oligodendrocytes. Neuron.

[12] Meyer M, Matsuoka I, Wetmore C, Olson L, Thoenen H (1992). Enhanced synthesis of brain-derived neurotrophic factor in the lesioned peripheral nerve different mechanisms are responsible for the regulation of BDNF and NGF mRNA. J Cell Biol.

[13] Costa RD, Mendonca VA, Penido RA, Lyon S, Costa AMDD, Costa MD (2011). Study of the profile of the neurotrophin BDNF in new leprosy cases before, during and after multidrug therapy. Arq Neuropsiquiatr.

[14] Chan JR, Cosgaya JM, Wu YJ, Shooter EM (2001). Neurotrophins are key mediators of the myelination program in the peripheral nervous system. Proc Natl Acad Sci USA.

[15] Trombone AP, Pedrini SC, Diorio SM, Belone AFF, Fachin LRV, Nascimento DC (2014). Optimized protocols for Mycobacterium leprae strain management frozen stock preservation and maintenance in athymic nude mice. J Vis Exp.

[16] Chan JP, Uong J, Nassiri N, Gupta R (2019). Lessons from leprosy peripheral neuropathies and deformities in chronic demyelinating diseases. J Hand Surg Am.

[17] Save MP, Shetty VP, Shetty KT (2009). Hypophosphorylation of NF-H and NF-M subunits of neurofilaments and the associated decrease in KSPXK kinase activity in the sciatic nerves of Swiss white mice inoculated in the foot pad with Mycobacterium leprae. Lepr Rev.

[18] Reichardt LF (2006). Neurotrophin-regulated signalling pathways. Philos Trans R Soc Lond B Biol Sci.

[19] Aarao TLS, de Sousa JR, Falcao ASC, Falcao LFM, Quaresma JAS (2018). Nerve growth factor and pathogenesis of leprosy review and update. Front Immunol.

[20] Mata M, Alessi D, Fink DJ (1990). S100 is preferentially distributed in myelin-forming Schwann cells. J Neurocytol.

[21] Jessen KR, Mirsky R, Lloyd AC (2015). Schwann cells development and role in nerve repair. Cold Spring Harb Perspect Biol.

[22] Fabrizi C, Kelly BM, Gillespie CS, Schlaepfer WW, Scherer SS, Brophy PJ (1997). Transient expression of the neurofilament proteins NF-L and NF-M by Schwann cells is regulated by axonal contact. J Neurosci Res.

[23] Sun Q, Tang DD, Yin EG, Wei LL, Chen P, Deng SP (2018). Diagnostic significance of serum levels of nerve growth factor and brain derived neurotrophic factor in diabetic peripheral neuropathy. Med Sci Monit.

[24] Hou X, Liang Q, Wu Y (2013). Transplantation of Schwann cells co-cultured with brain-derived neurotrophic factor for the treatment of experimental autoimmune neuritis. J Neuroimmunol.

[25] Richner M, Ulrichsen M, Elmegaard SL, Dieu R, Pallesen LT, Vaegter CB (2014). Peripheral nerve injury modulates neurotrophin signaling in the peripheral and central nervous system. Mol Neurobiol.

[26] Yin Q, Kemp GJ, Yu LG, Wagstaff SC, Frostick SP (2001). Expression of Schwann cell-specific proteins and low-molecular-weight neurofilament protein during regeneration of sciatic nerve treated with neurotrophin-4. Neuroscience.

[27] Rodriguez-Pena A, Botana M, Gonzalez M, Requejo F (1995). Expression of neurotrophins and their receptors in sciatic nerve of experimentally diabetic rats. Neurosci Lett.

[28] McKenzie IA, Biernaskie J, Toma JG, Midha R, Miller FD (2006). Skin-derived precursors generate myelinating Schwann cells for the injured and dysmyelinated nervous system. J Neurosci.

[29] Saulite L, Vavers E, Zvejniece L, Dambrova M, Riekstina U (2018). The differentiation of skin mesenchymal stem cells towards a Schwann cell phenotype impact of sigma-1 receptor activation. Mol Neurobiol.

[30] Liu N, Varma S, Tsao D, Shooter EM, Tolwani RJ (2007). Depleting endogenous neurotrophin-3 enhances myelin formation in the Trembler-J mouse, a model of a peripheral neuropathy. J Neurosci Res.

